# SDL Index Predicts Stroke-Associated Pneumonia in Patients After Endovascular Therapy

**DOI:** 10.3389/fneur.2021.622272

**Published:** 2021-02-16

**Authors:** Bowei Zhang, Wenbo Zhao, Chuanjie Wu, Longfei Wu, Chengbei Hou, Kara Klomparens, Yuchuan Ding, Chuanhui Li, Jian Chen, Jiangang Duan, Yunzhou Zhang, Hong Chang, Xunming Ji

**Affiliations:** ^1^Department of Neurology, Xuanwu Hospital of Capital Medical University, Beijing, China; ^2^Center for Evidence-Based Medicine, Xuanwu Hospital of Capital Medical University, Beijing, China; ^3^Department of Neurosurgery, Wayne State University School of Medicine, Detroit, MI, United States; ^4^Department of Emergency, Xuanwu Hospital of Capital Medical University, Beijing, China; ^5^Department of Neurosurgery, Xuanwu Hospital of Capital Medical University, Beijing, China

**Keywords:** stroke-associated pneumonia, endovascular therapy, lymphopenia, acute ischemic stroke, prediction scoring system

## Abstract

**Objective:** This study aimed to develop and validate a novel index to predict SAP for AIS patients who underwent endovascular treatment.

**Methods:** A study was conducted in an advanced comprehensive stroke center from January 2013 to December 2019 aiming to develop and validate a novel index to predict SAP for AIS patients who underwent endovascular treatment. This cohort consisted of a total of 407 consecutively registered AIS patients who underwent endovascular therapy, which was divided into derivation and validation cohorts. Multiple blood parameters as well as demographic features, vascular risk factors, and clinical features were carefully evaluated in the derivation cohort. The independent predictors were obtained using multivariable logistic regression. The scoring system was generated based on the β-coefficients of each independent risk factor.

**Results:** Ultimately, a novel predictive model: the SDL index (stroke history, dysphagia, lymphocyte count < 1.00 × 10^3^/μL) was developed. The SDL index showed good discrimination both in the derivation cohort (AUROC: 0.739, 95% confidence interval, 0.678–0.801) and the validation cohort (AUROC: 0.783, 95% confidence interval, 0.707–0.859). The SDL index was well-calibrated (Hosmer–Lemeshow test) in the derivation cohort (*P* = 0.389) and the validation cohort (*P* = 0.692). We therefore divided our population into low (SDL index = 0), medium (SDL index = 1), and high (SDL index ≥ 2) risk groups for SAP. The SDL index showed good discrimination when compared with two existing SAP prediction models.

**Conclusions:** The SDL index is a novel feasible tool to predict SAP risk in acute ischemic stroke patients post endovascular treatment.

## Introduction

Stroke-associated pneumonia (SAP) is one of the most common complications in stroke patients occurring in 5–26% in general (1–5). However, acute ischemic stroke patients caused by large vessel occlusion often accompanied by greater stroke severity, the prevalence of SAP would be much higher. For large hemisphere infarction, the prevalence of SAP can increase to about 50% (6). Endovascular therapy, especially mechanical thrombectomy, has been the recommended therapy because it demonstrates a high rate of vessel recanalization and improved functional outcomes for these patients. SAP has been well-known for worsening patient outcomes, increasing disability and increasing mortality in stroke patients (7–10). It has been of vital significance to investigate the predictive methods of SAP for this population in order to identify patients who are at greater risk so that proper treatment and care can be initiated.

However, the existing predictive models which are mainly based on clinical features such as age, dysphagia, stroke severity, diabetes mellitus, atrial fibrillation, etc. (2, 7, 11–15), were neither derived nor validated in patients who underwent endovascular therapy in particular. What's more, emerging evidences have shown that multiple blood parameters including their ratios such as neutrophil to lymphocyte ratio (NLR), platelet to lymphocyte ratio (PLR), and monocyte to lymphocyte ratio (MLR) as markers of inflammation, are correlated with pneumonia, outcome and even SAP as well (16–24). Therefore, we aimed to achieve two goals in this study: (1) to investigate the independent predictors of SAP in AIS patients who underwent endovascular treatment in particular to generate and validate an applicable predictive model; (2) to compare the predictive value to prior validated scores in this population.

## Materials and Methods

### Study Design and Population

We performed a retrospective analysis of a prospective collected database which contains all consecutive patients with AIS receiving endovascular therapy in an advanced comprehensive stroke center (Capital Medical University Xuanwu Hospital, Beijing, China) from January 2013 to December 2019 (*n* = 786). This registry had been approved by the Ethics Committee of Capital Medical University Xuanwu Hospital and written informed consent was obtained by all the patients or their legal representatives on admission.

**Figure 1:**
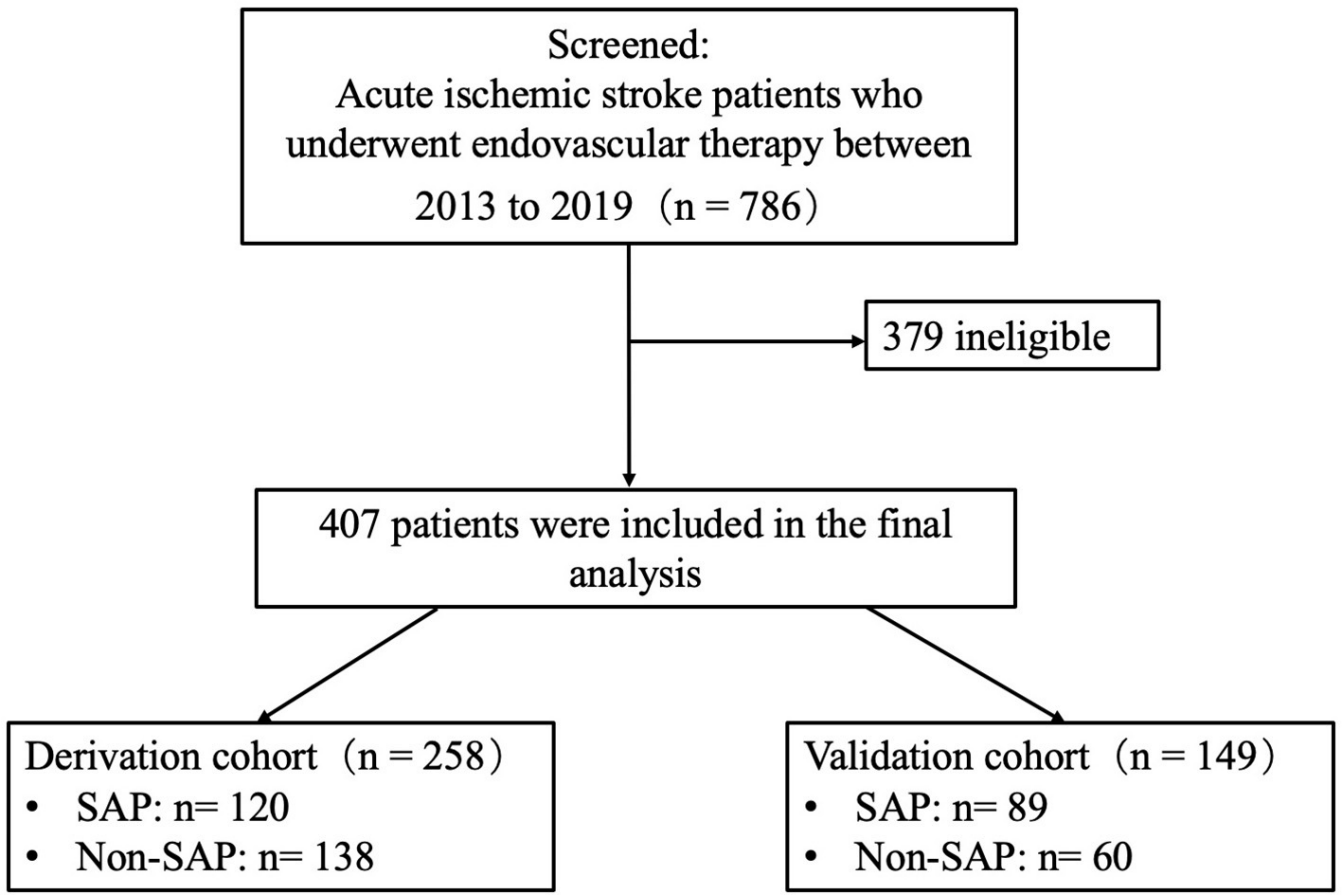
A study flowchart. Of the 786 patients screened, 407 patients were included in the final analysis. 258 patients were allocated to a derivation cohort and 149 patients in the validation cohort.

Eligibility for this study were (1) AIS patients caused by large vessel occlusion who underwent endovascular treatment (intra-arterial rt-PA, mechanical clot disruption or retrieval, with or without a combination of permanent stenting) under local anesthesia (with the purpose of avoiding the influence of ventilator-related pneumonia), (2) availability blood examinations results completed within 24 h from the time of symptom onset or last known healthy, and (3) confirmation of the diagnosis of SAP or non-SAP within 7 days of stroke onset. Four hundred and seven patients were included in the final analysis (*n* = 407). The therapeutic regimens of endovascular therapy followed the recommendation of guidelines (25–27). All the patients were admitted in the stroke unit of this comprehensive stroke center and the management is consistent. Eligible patients from January 2013 to December 2018 (*n* = 258) were allocated to a derivation cohort and the patients in the year of 2019 (*n* = 149) to a validation cohort. A study flowchart was shown as Figure 1.

### Data Collection

A total of 258 patients were ultimately included in the derivation cohort. Baseline demographic characteristics and vascular risk factors were evaluated including age, sex, hypertension, diabetes mellitus, atrial fibrillation, hyperlipidemia, stroke history, coronary artery disease, smoking, and excess alcohol consumption. Clinical features including initial National Institutes of Health Stroke Scale (NIHSS)-scores, admission Alberta Stroke Program Early CT Score (ASPECTS) score or Posterior Circulation Alberta Stroke Program Early CT Score (pc-ASPECTS), dysphagia, the time interval from symptoms onset to admission, procedural details and recanalization, admission serum glucose level and blood pressure levels as well as stroke etiology subtypes were all recorded. A total of 149 patients were ultimately included in the validation cohort. The following baseline factors were compared with the derivation cohort after the predictive model was developed: age, sex, stroke severity represented by initial NIHSS score, dysphagia, atrial fibrillation, stroke history, lymphocyte count as well as the comparison of two existing predictive scores including A^2^DS^2^ [age, atrial fibrillation, dysphagia, sex, stroke severity (National Institutes of Health Stroke Scale)] and ISAN [prestroke Independence (modified Rankin scale), Sex, Age, National Institutes of Health Stroke Scale].

Blood parameters in this analysis were obtained within 30 min after admission. The blood parameters we analyzed including white blood cell (WBC), neutrophils, lymphocytes, monocytes, hemoglobin, platelet, fibrinogen, d-dimer, NLR, PLR, and MLR. Venous blood samples were drawn into standardized tubes containing an anticoagulant (EDTA) at admission and stored at room temperature. The value of white blood cell (WBC) count, neutrophil, lymphocyte, monocyte, hemoglobin, and platelet counts were from the complete blood tests determined using Sysmex XE-5000 autoanalyzer within 1 h after sample collection. In the meantime, fibrinogen and d-dimer level were from the coagulation blood test determined using Stago Revolution autoanalyzer. PLR, NLR, and MLR were calculated by the ratio of the absolute counts of platelet, neutrophil or monocyte to lymphocyte. Dysphagia was evaluated by a bedside swallowing tests performed at our hospital within the 1st day after admission. SAP was diagnosed by the specially trained treating physician who work in our advanced comprehensive stroke center based on the combination of clinical symptoms, radiological findings, and pathogen detection according to the modified Centers for Disease Control and Prevention criteria of stroke-associated pneumonia (28). Stroke-associated pneumonia (SAP) is the recommended terminology for the spectrum of lower respiratory tract infections within the first 7 days after stroke onset. All the SAPs diagnosed in this study were reaching the diagnosis of definite SAP according to the consensus. Pneumonia before stroke was not considered as SAP. Clinical outcome was evaluated according to the modified Rankin Score (mRS) at 90 days through telephone follow-up or clinical visits. Favorable functional outcome was defined as mRS 0–2 and unfavorable functional outcome was defined as mRS 3–6. The 10-point A^2^DS^2^ [age, atrial fibrillation, dysphagia, sex, stroke severity (National Institutes of Health Stroke Scale)] score and the 21-point ISAN [prestroke Independence (modified Rankin scale), Sex, Age, National Institutes of Health Stroke Scale] score were assigned as described previously (12, 29).

### Intervention

Patients were treated as soon as possible after the symptom's onset. Only the neurointerventionists who are specially trained in the technique of performing extracranial and intracranial stents qualified for endovascular treatment performed the interventions. All procedures were performed using the groin puncture approach. The specific intervention strategies including intra-arterial recombinant tissue plasminogen activator (rt-PA), the type of stent retrievers, with or without a combination of permanent stenting and other devices were decided by the neurointerventionists according to the situation. Recanalization results were assessed by the Modified Thrombolysis in Cerebral Infarction (mTICI) Scale. A post-treatment mTICI score of 2b or 3 was considered as successful recanalization. The post-surgery management was consistent among all patients.

### Statistical Analysis

Data was processed using SPSS 23.0 for Mac (IBM, Chicago, IL, USA) and STATA/SE 15.1(StataCorp, Texas, USA). Continuous variables were exhibited by mean ± SD (normal distribution) or median, IQR (skewed distribution), which was analyzed by independent *t*-test or Mann–Whitney *U*-test for continuous variables, and the Chi-Square test or Fisher's exact test were used for categorical variables. Distribution normality was tested by the Kolmogorov–Smirnov test. The predictive power of lymphocyte count was compared using receiver operating characteristic (ROC) curve and the area under the curve and the optimal cut-off point were calculated. Multivariable analysis was performed using logistic regression models, it was adjusted for other variables that were selected from univariate analyses and previous studies. The final multivariable model was based on stepwise backward estimation to remove nonsignificant variables from the model. The score system was generated according to the β-coefficients from each component in the final multivariable analysis. The SDL index was validated by assessing discrimination and calibration. Area under the receiver operating characteristic curve (AUROC) was used to measure the discrimination. An AUROC of 0.5 indicates no ability, and an AUROC of 1.0 indicates perfect discrimination to differentiate patients with SAP and without SAP. Hosmer–Lemeshow goodness-of-fit test was used to measure calibration. A *P* < 0.05 was considered significant for all analyses.

### Data Availability

Any data not published within the article will be shared in anonymized form by request from any qualified investigator.

## Results

### Characteristics of Patients in the Derivation Cohort

From January 2013 to December 2018 a total of 546 patients were consecutively screened, and 258 patients who underwent local anesthesia with complete data were included. The median age was 63 (interquartile range [IQR], 55–72) years, and 169 (65.5%) of the patients were men. The median initial NIHSS score of this cohort was 14 (10–18). Successful recanalization was achieved in 240 cases (93%). SAP occurred in 120 (46.5%) patients.

Baseline clinical characteristics between groups with and without SAP as well as potential blood parameters are presented in Tables 1, 2. In the univariate analysis, the SAP group presented with significantly older age (*p* = 0.003), lower Glasgow Coma score (GCS) score (*p* = 0.001), higher proportion of dysphagia (*p* < 0.001), higher baseline systolic blood pressure (*p* = 0.031), higher rates of previous stroke history (*p* = 0.001) and lower rates of efficient recanalization (*p* = 0.001). The SAP group also had a higher proportion of diabetes mellitus (*p* = 0.051). Among the measured blood parameters, neutrophil (*p* = 0.001), NLR (*p* < 0.001), PLR (*p* < 0.001), and MLR (*p* = 0.007) and d-dimer (*p* = 0.013) were significantly higher in SAP group, while lymphocyte count (*p* < 0.001) was significantly lower in the SAP group compared to the non-SAP group [neutrophils: 8.5 (6.6–10.3) vs. 7.4 (6.3–9.5); PLR: 227 (171–312) vs. 167 (120–252); NLR: 10.5 (5.8–15.1) vs. 6.6 (4.0–9.2); MLR: 0.43 (0.29–0.67) vs. 0.36 (0.25–0.51); lymphocyte: 0.9 (0.6–1.1) vs. 1.2 (0.9–1.6)].

**Table 1:** Baseline characteristics of patients with SAP and non-SAP in acute ischemic stroke underwent endovascular treatment.

**Characteristics**	**All (*N* = 258)**	**SAP (*N* = 120)**	**Non-SAP (*N* = 138)**	***P-*value**
**Demographics**				
Age, median [IQR], y	63 (55–72)	67 (57–76)	62 (54–70)	0.003
Male, *n* (%)	169 (65.5)	78 (65.0)	91 (65.9)	0.874
GCS median [IQR]	13(9–15)	11 (8–14)	14 (10–15)	0.001
Dysphagia, *n* (%)	105 (40.7)	70 (58.3)	35 (25.4)	<0.001
Initial NIHSS score median [IQR]	14 (10–18)	17 (12–22)	12 (9–16)	<0.001
ASPECTS (or pc-ASPECTS) median [IQR]	9 (8–10)	9 (7–10)	9 (8–10)	0.204
Systolic blood pressure, median [IQR], mmHg	140 (127–154)	144 (130–158)	138 (122–150)	0.031
Diastolic blood pressure, median [IQR], mmHg	80 (72–90)	82 (75–90)	80 (71.5–90)	0.097
Admission blood glucose, median [IQR], mmol/L	7.1 (5.9–9.3)	7.9 (6.1–10.1)	6.8 (5.8–8.7)	0.196
Anterior circulation stroke, *n* (%)	201 (77.9)	91 (75.8)	110 (79.7)	0.454
Posterior circulation stroke, *n* (%)	57 (22.1)	29 (24.2)	28 (20.3)	
**Vascular risk factors**, ***n*** **(%)**				
hypertension	176 (68.2)	88 (73.3)	88 (63.8)	0.100
Diabetes mellitus	69 (26.7)	39 (32.5)	30 (21.7)	0.051
Atrial fibrillation	68 (26.4)	37 (30.8)	31 (22.5)	0.128
Hyperlipidemia	60 (23.3)	28 (23.3)	32 (23.2)	0.978
Stroke history	55 (21.3)	37 (30.8)	18 (13.0)	0.001
Coronary artery disease	38 (14.7)	21 (17.5)	17 (12.3)	0.241
Smoking	110 (42.6)	47 (39.2)	63 (45.7)	0.293
Excess alcohol consumption	81 (31.4)	38 (31.7)	43 (31.2)	0.930
**Stroke etiology**, ***n*** **(%)**				
Large artery atherosclerosis	181 (70.2)	81 (67.5)	102 72.3)	0.400
cardioembolism	60 (23.3)	32 (26.7)	28 (19.9)	0.361
Other or undetermined	17 (6.6)	4 (3.3)	11 (7.8)	0.389
**Time intervals, min [IQR]**				
From the symptom's onset to admission	260 (173–360)	247 (180–360)	267 (173–354)	0.619
**Operational details**, ***n*** **(%)**				
Treatment with intravenous alteplase	96 (37.2)	45 (37.5)	51 (37.0)	0.928
intra-arterial thrombolysis	42 (16.3)	18 (15.0)	24 (17.4)	0.604
Stent-retrieving	209 (81.0)	101 (84.2)	108 (78.3)	0.229
Permanent stenting	65 (25.2)	30 (25.0)	35 (25.4)	0.947
**Recanalization**, ***n*** **(%)**				
TICI 2b or 3	240 (93.0)	106 (88.3)	134 (97.1)	0.001

*mTICI, Modified Thrombolysis in Cerebral Infarction; IQR, interquartile range; NIHSS, National Institutes of Health Stroke Scale; GCS, Glasgow Coma score*.

**Table 2:** Baseline levels of blood parameter of patients with SAP and Non-SAP in acute ischemic stroke underwent endovascular treatment.

**Blood parameters**	**All (*N* = 258)**	**SAP (*N* = 120)**	**Non-SAP (*N* = 138)**	***P-*value**
WBC, median [IQR], × 10^3^/μL	9.5 (7.9–11.4)	9.8 (7.9–11.7)	9.3 (8.0–11.0)	0.141
Neutrophil, median [IQR], × 10^3^/μL	7.9 (6.5–9.9)	8.5 (6.6–10.3)	7.4 (6.3–9.5)	0.011
Lymphocyte, median [IQR], × 10^3^/μL	1.0 (0.7–1.5)	0.9 (0.6–1.1)	1.2 (0.9–1.6)	<0.001
Monocyte, median [IQR], × 10^3^/μL	0.4 (0.3–0.6)	0.4 (0.3–0.5)	0.5 (0.3–0.6)	0.191
Hemoglobin, g/dL [IQR]	134 (121–145)	131 (121–143)	137 (121–146)	0.115
Platelet, median [IQR], × 10^3^/μL	200 (167–232)	191 (159–225)	207 (173–234)	0.532
Fibrinogen, median [IQR], g/L	3.0 (2.6–3.5)	3.1 (2.7–3.6)	3.0 (2.6–3.5)	0.413
D-dimer, median [IQR], mg/L	1.2 (0.5–3.7)	1.3 (0.6–3.1)	1.0 (0.4–2.2)	0.013
NLR, median [IQR]	7.8 (4.9–13.0)	10.7 (5.8–15.0)	6.5 (4.0–9.1)	<0.001
PLR, median [IQR]	195 (134–279)	229 (171–319)	167 (120–244)	<0.001
MLR, median [IQR]	0.38 (0.26–0.57)	0.43 (0.29–0.67)	0.36 (0.24–0.51)	0.007

*WBC, White blood cell; IQR, interquartile range; NLR, Neutrophil-to-lymphocyte ratio; PLR, Platelet-to-lymphocyte ratio; MLR, Monocyte-to-lymphocyte ratio*.

Multivariable logistic regression analysis was used to evaluate the independent predictors to SAP (Table 3). The lymphocyte count (adjusted OR = 0.303, 95% CI, 0.124–0.741, *p* = 0.009) was the only blood parameter measured that remained to be significant after adjusting for confounders (Supplementary Material). Additionally, dysphagia (adjusted OR = 2.768, 95% CI, 1.180–6.494, *p* = 0.019*) and previous stroke history (adjusted OR = 4.482, 95% CI, 1.720–11.678, *p* = 0.002*) also remained significant as independent predictors. ROC curve was then employed to evaluate the predictive value of the lymphocyte count, dysphagia and stroke history (Supplementary Material). The area under curve (AUC) and the optimal cut-off value were calculated.

**Table 3:** Multivariable analysis of possible predictors of SAP.

	**Adjusted OR**	**95% CI**	***P-*value**
Age, y [IQR]	1.013	0.974–1.053	0.515
Male, *n* (%)	1.263	0.569–2.800	0.566
Systolic blood pressure, mmHg [IQR]	1.007	0.989–1.025	0.431
Diabetes mellitus, *n* (%)	0.968	0.409–2.291	0.940
Atrial fibrillation, *n* (%)	1.257	0.512–3.089	0.618
Stroke history, *n* (%)	4.482	1.720–11.678	0.002^*^
Dysphagia, *n* (%)	2.768	1.180–6.494	0.019^*^
Initial NIHSS score [IQR]	1.044	0.970–1.125	0.251
GCS [IQR]	0.975	0.820–1.158	0.770
Recanalization, *n* (%)	0.295	0.029–3.017	0.303
Lymphocyte count [IQR]	0.303	0.124–0.741	0.009^*^

*IQR, interquartile range; NIHSS, National Institutes of Health Stroke Scale; GCS, Glasgow Coma score*.

* *Adjusted with factors P < 0.10 in univariate analysis (age, systolic blood pressure, diabetes mellitus, dysphagia, stroke history, initial NIHSS score, GCS, and recanalization) as well as gender and atrial fibrillation which were previously reported risk factor (11, 12)*.

### Development of the SDL Index

Based on the β-coefficient of the final multivariable logistic regression model with stepwise backward variable selection (Table 4), one point was assigned to each of independent predictors (dysphagia presence, previous stroke history, and lymphocyte count < 1.00 × 10^3^/μL) (Supplementary Material). Therefore, the SDL index was generated (Table 5).

**Table 4:** Final multivariable logistic regression analysis of predictors of SAP.

	**β-Coefficient**	**SE**	**Adjusted OR**	**95%CI**	***P-*value**
Stroke history	1.357	0.380	3.885	1.847–8.175	<0.001
Dysphagia	1.383	0.301	3.985	2.209–7.190	<0.001
Lymphocyte count < 1.00 × 10^3^/μL	1.226	0.309	3.407	1.861–6.239	<0.001

*Variables were eliminated using backward elimination procedure*.

**Table 5:** Components of *SDL* index.

**Components**	**SDL index**
Stroke history	
With	+1
Without	0
Dysphagia	
Present	+1
Absent	0
Lymphocyte count	
<1.00 × 10^3^/μL	+1
≥1.00 × 10^3^/μL	0
Total *SDL* index	0–3

*SDL indicates Stroke history, Dysphagia, Lymphocyte count < 1.00 × 10^3^/μL*.

### SAP and 3-Month Outcomes in the Derivation Cohort

**Figure 2:**
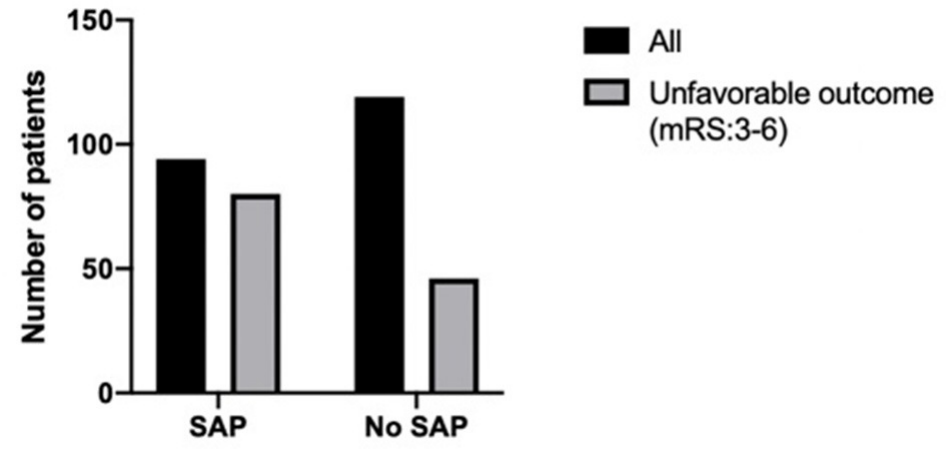
Differences in 3-month outcome according to patients with SAP or non-SAP. Among 94 patients with SAP, 80 patients got unfavorable outcome (mRS 3–6). Among 119 non-SAP patients, 46 patients got unfavorable outcome. SAP is significantly associated with unfavorable outcome at 3-month (*p* < 0.001).

Three month mRS scores were available in 213 patients. Among the 94 patients with SAP, 80 patients demonstrated an unfavorable outcome (mRS 3–6). Among the 119 non-SAP patients, 46 patients demonstrated an unfavorable outcome. This data shows that SAP is significantly associated with unfavorable outcome at 3-month (*p* < 0.001) (Figure 2).

### Validation of the SDL Index and the Comparison With the Existing Scores

**Figure 3:**
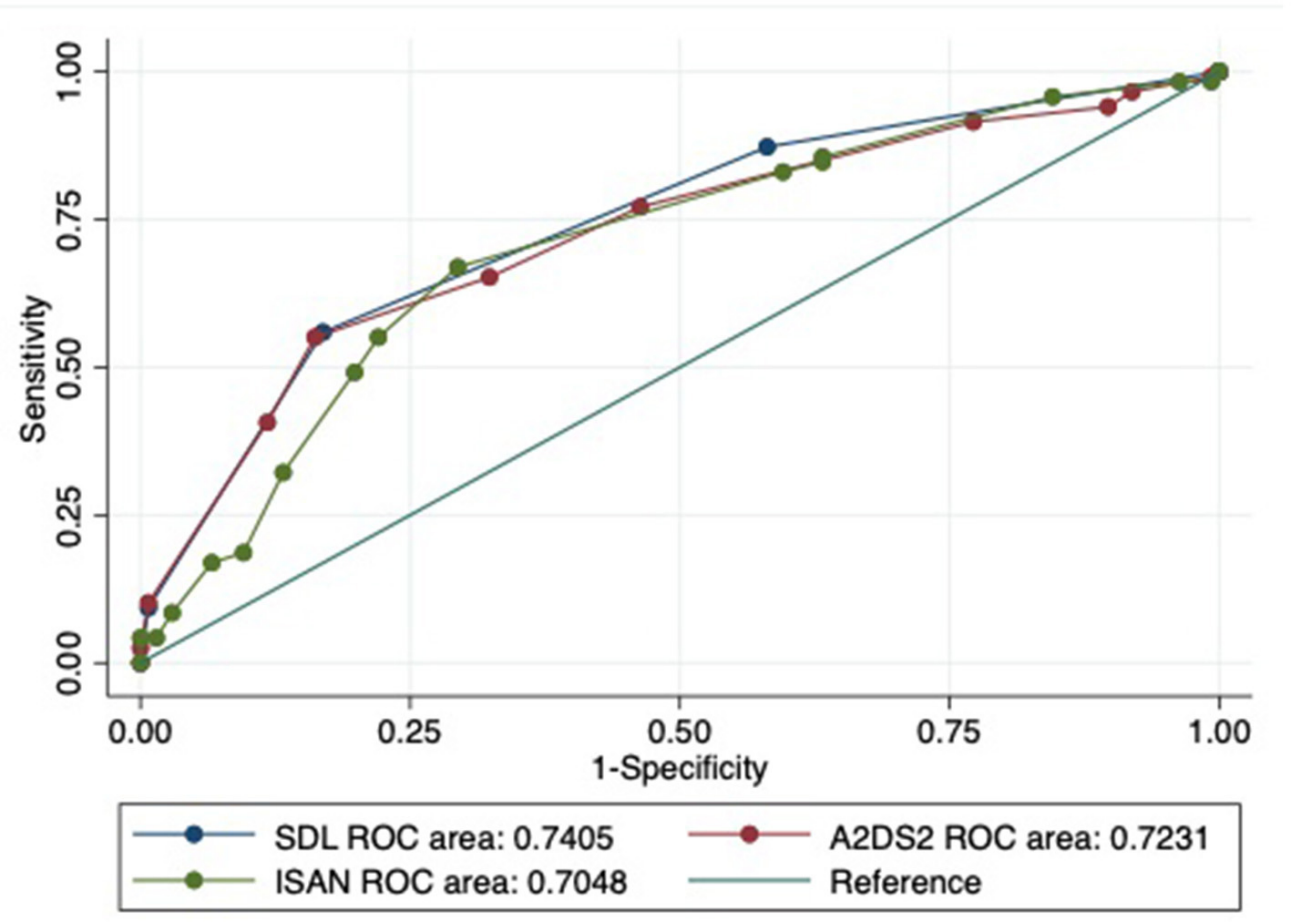
Validation of discrimination in the derivation cohort. ROC, receiver operating characteristic curve; SDL indicates *S*troke history, *D*ysphagia, *L*ymphocyte count < 1.00 × 10^3^/μL; A^2^DS^2^ indicates *a*ge, *a*trial fibrillation, *d*ysphagia, *s*ex, *s*troke severity (National Institutes of Health Stroke Scale); ISAN, prestroke *I*ndependence (modified Rankin scale), *S*ex, *A*ge, *N*ational Institutes of Health Stroke Scale.

**Figure 4:**
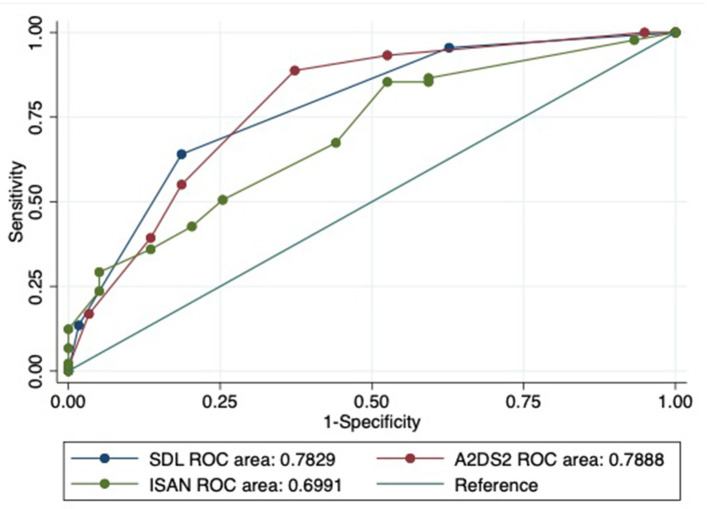
Validation of discrimination in the validation cohort. ROC, receiver operating characteristic curve; SDL indicates *S*troke history, *D*ysphagia, *L*ymphocyte count < 1.00 × 10^3^/μL; A^2^DS^2^ indicates *a*ge, *a*trial fibrillation, *d*ysphagia, *s*ex, *s*troke severity (National Institutes of Health Stroke Scale); ISAN, prestroke *I*ndependence (modified Rankin scale), *S*ex, *A*ge, *N*ational Institutes of Health Stroke Scale.

The comparison of the baseline characteristics as well as the predictive scores including SDL index, A^2^DS^2^ and ISAN scores between the derivation and the validation cohort were shown (Table 6). The discrimination of the SDL index (AUROC) was 0.739 (95% confidence interval: 0.678–0.801) in the derivation cohort and 0.783 (95% confidence interval: 0.707–0.859) in the validation cohort. When compared with A^2^DS^2^ and ISAN score, SDL continued to show good performance (Table 7). The AUROC of SDL index was shown to be the highest among those 3 scores in the derivation cohort even hadn't reached to a statistical significance (*p* = 0.56) (Figure 3). And SDL index shared similar performance with A^2^DS^2^ score (*p* = 0.88) in the validation cohort which was non-significantly higher than ISAN score (*p* = 0.09) (Figure 4). The SDL index was well-calibrated (Hosmer–Lemeshow test) in the derivation cohort (*P* = 0.389) and the validation cohort (*P* = 0.692). As shown in the Table 7, when SDL ≥ 1, both the sensitivity value and negative predictive value were highest when compared with other scores; when SDL ≥ 2, both the specificity value and positive predictive value were highest when compared with other scores; worth to be mentioned, when SDL = 3, both the specificity value and the positive predictive value reached over 90% both in derivation and validation cohort.

**Table 6:** Comparison of the characteristics between the derivation and the validation cohort.

	**Derivation cohort (*n* = 258)**	**Validation cohort (*n* = 149)**	***P-*value**
Age, y [IQR]	63 (55–72)	65 (57–74)	0.359
Male, *n* (%)	169 (65.5)	106 (71.1)	0.272
Initial NIHSS score [IQR]	14 (10–18)	14 (11–18)	0.674
Dysphagia, *n* (%)	105 (40.7)	95 (63.8)	<0.001
Atrial fibrillation, *n* (%)	68 (26.4)	40 (26.8)	0.908
Stroke history, *n* (%)	55 (21.3)	34 (22.8)	0.804
Lymphocyte count × 10^3^/μL [IQR]	1.01 (0.70–1.45)	0.98 (0.69–1.45)	0.855
Lymphocyte count <1.00 × 10^3^/μL, *n* (%)	125 (48.4)	75 (50.3)	0.758
SDL = 0, *n* (%)	74 (28.7)	26 (17.4)	0.012
SDL = 1, *n* (%)	95 (36.8)	55 (36.9)	0.675
SDL = 2, *n* (%)	77 (29.8)	55 (36.9)	0.154
SDL = 3, *n* (%)	12 (4.7)	13 (8.7)	0.132
SDL index [IQR]	1 (0–2)	1(1–2)	0.006
A^2^DS^2^ score (12) [IQR]	5 (4–7.25)	6 (5–8)	0.001
ISAN score (29) [IQR]	8 (5–11)	9 (7–12)	0.117
SAP, *n* (%)	120 (46.5)	89 (59.7)	0.013

*IQR, interquartile range; NIHSS, National Institutes of Health Stroke Scale; SDL indicates Stroke history, Dysphagia, Lymphocyte count < 1.00 × 10^3^/μL; A^2^DS^2^ indicates age, atrial fibrillation, dysphagia, sex, stroke severity (National Institutes of Health Stroke Scale); ISAN, prestroke Independence (modified Rankin scale), Sex, Age, National Institutes of Health Stroke Scale*.

**Table 7:** Discrimination of SDL index compared with 2 prior scores in predicting SAP.

	**AUROC**	**95% CI**	***P-*value**	**Cut-off**	**Sensitivity**	**Specificity**	**PPV**	**NPV**
**Derivation cohort (n=258)**								
SDL index	0.739	0.678–0.801	<0.001					
SDL ≥ 1					0.867	0.428	0.568	0.787
SDL ≥ 2					0.550	0.833	0.741	0.680
SDL = 3					0.092	0.993	0.917	0.557
A^2^DS^2^ score (12)	0.726	0.663–0.789	<0.001	6	0.650	0.681	0.639	0.691
ISAN score (29)	0.701	0.636–0.766	<0.001	8	0.817	0.413	0.547	0.722
**Validation cohort (*****n*** **=** **149)**								
SDL index	0.783	0.707–0.859	<0.001					
SDL ≥ 1					0.955	0.383	0.691	0.885
SDL ≥ 2					0.640	0.817	0.838	0.605
SDL = 3					0.135	0.983	0.923	0.434
A^2^DS^2^ score (12)	0.789	0.711–0.867	<0.001	6	0.888	0.633	0.782	0.791
ISAN score (29)	0.699	0.614–0.784	<0.001	8	0.854	0.483	0.710	0.690

*AUROC, area under the receiver operating characteristic curve; SDL indicates Stroke history, Dysphagia, Lymphocyte count < 1.00 × 10^3^/μL; A^2^DS^2^ indicates age, atrial fibrillation, dysphagia, sex, stroke severity (National Institutes of Health Stroke Scale); ISAN, prestroke Independence (modified Rankin scale), Sex, Age, National Institutes of Health Stroke Scale; PPV, positive predictive value; NPV, negative predictive value*.

### SDL Index Risk Stratifications

**Figure 5:**
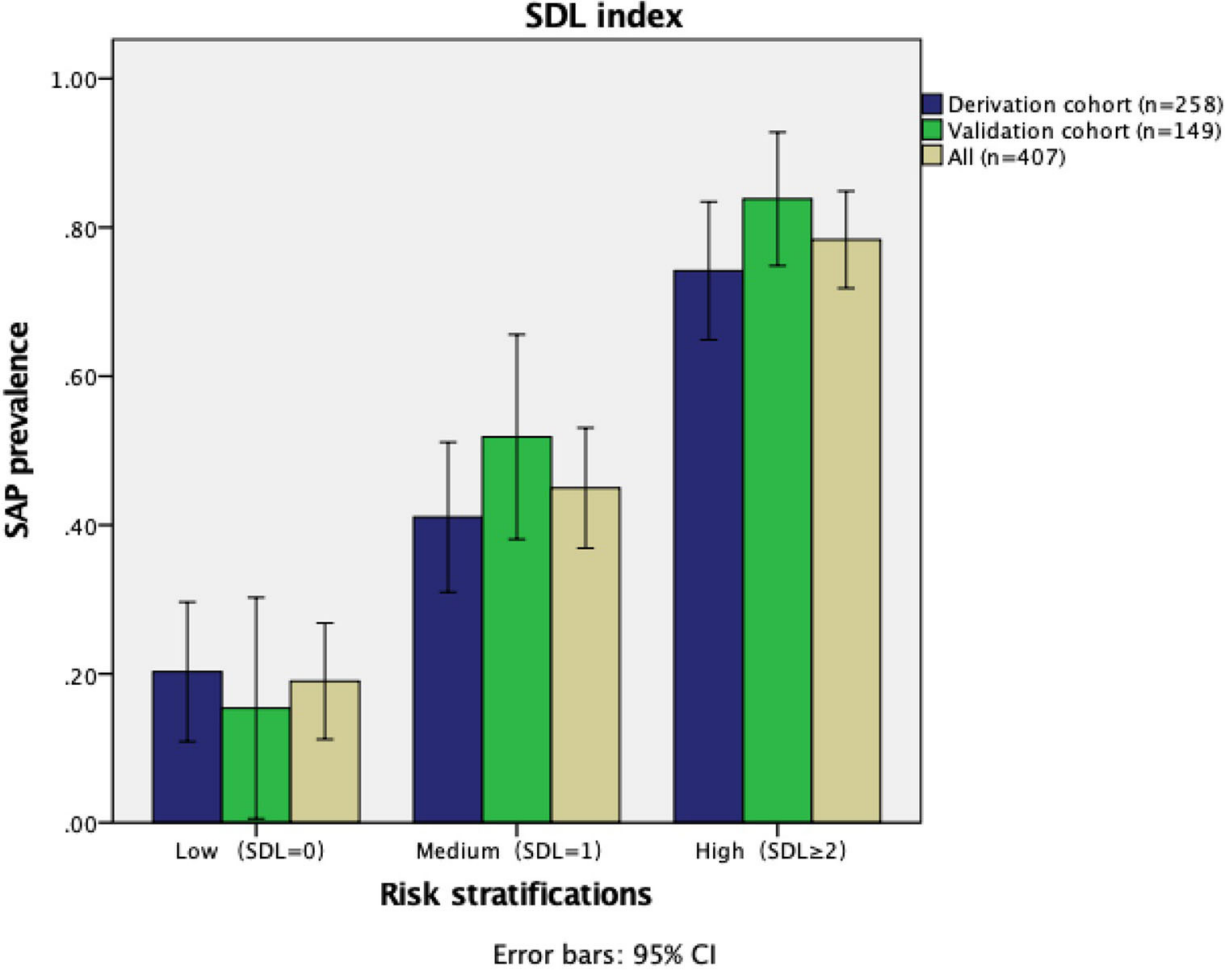
The prevalence of SAP in the derivation, validation cohort, and in total. The error bars indicated 95% confidence interval for the prevalence of SAP in each category. SDL indicates *S*troke history, *D*ysphagia, *L*ymphocyte count < 1.00 × 10^3^/μL.

Based on the score of SDL index, we divided our population into low (SDL index = 0), medium (SDL index = 1), high (SDL index ≥ 2) risk group for SAP. The prevalence of SAP in the low-risk group was 20.3% (derivation cohort), 15.4% (validation cohort), and 19% (combined). The prevalence of SAP in the medium-risk group was 41.1% (derivation cohort), 50.9% (validation cohort), and 44.7% (combined), respectively. And the prevalence of SAP in the high-risk group was 74.2% (derivation cohort), 83.8% (validation cohort), and 78.3% (combined) (Figure 5).

## Discussion

In this study, we aimed to develop a simple, feasible tool to predict SAP particularly for the increasing population of acute ischemic stroke patients who underwent endovascular treatment. Since endovascular therapy has become a global trend for AIS treatment, there is an urgent need for a predictive model specially designed for this population. Moreover, the time restrictions are extremely prominent in emergency departments or acute stroke units, physicians always need to make quick judgments with a limited amount of time and information. Therefore, we believe that a simple and well-validated score will be largely preferred over a complex model which is difficult to memorize and calculate in this situation.

The SDL index was developed based on three significant risk factors for SAP: stroke history, dysphagia and lymphopenia, which all have easy availability in the clinical field. SAP incidence significantly indicated unfavorable outcome at 3-month follow up in our study population. The discrimination and calibration of this index were well-validated. According to the scores of SDL index, three risk categories (low, medium, and high) of SAP were developed. The developed SDL index was then compared to existing SAP indexes including A^2^DS^2^, ISAN (12, 29). Among these 3 scoring systems, the SDL index is the simplest predictive model and performed well in comparison. Another index for SAP, the AIS-APS score, does exist; however, it was not possible to compare to the SDL index because AIS-APS includes COPD and current smoking status which were not recorded in this study (11).

As time is a limiting factor in many clinical environments, a simple predictive model will be preferred over a complicated scale allowing physicians to make quick judgments about the patient's current needs and status in the real working situation. In addition to time constraints, emergency departments also deal with a lack of definite medical history. Therefore, a valid and simple score will be a better option. Compared with other existing scores, the SDL index developed in this study with its simplicity and time-saving nature that there is even no need to memorize or calculate especially. SDL index has a major advantage in this sense. What's more, for those identified as high-risk patients, some therapeutic measures (prophylactic antibiotics, immunomodulators, inclusion in clinical trials) might be considered in the future.

In this study, we also particularly considered the result of blood examination as a component of the predictive model due to the importance of the stroke-induced immunodepression in predisposing SAP. Stroke-induced immunodepression is manifested by lymphopenia, monocyte deactivation and a shift from Th1 to Th2 cytokine production in the peripheral immune system (30–34). The underlying mechanisms are involved with the hypothalamic-pituitary-adrenal (HPA) axis, sympathetic and parasympathetic innervation (35). After comparing with multiple blood parameters, our study results particularly revealed lymphopenia to be a significant predictor for SAP and thus was included as a component of the developed SDL index. According to previous studies, lymphopenia is more common in severe stroke which is rather immediate and short-lived compared to leukocytes and monocytes. The role of which in stroke has been more and more realized over the decades (36–41). Most of the AIS patients who underwent endovascular therapy have suffered large vessel occlusion. The time window for endovascular treatment in this situation is limited. This allows a short time frame after symptoms onset to obtain blood samples. For this reason, lymphopenia is more prominent in acute ischemic stroke patients who underwent endovascular therapy.

Patients with SAP also had significant older age, greater stroke severity, higher systolic blood pressure, increased incidence of dysphagia, increased number of previous stroke events, and a more impaired level of consciousness than patients without SAP in our study population. These aspects were all found to be risk factors for SAP consistent with previous studies (2–5, 21, 42). However, among these factors only stroke history and presence of dysphagia remained significant contributors after adjusting for confounders in the multivariable logistic regression model. Surprisingly, to some extent, higher NIHSS score and impaired level of consciousness didn't remain as independent factors in the multivariable regression model. After consideration, we can give explanations as follows. Higher NIHSS score not remaining significant is potentially due to the greater stroke severity seen in AIS patients who undergo endovascular treatment. High initial NIHSS score or lymphopenia can be used to represent stroke severity. The impaired level of consciousness not remaining significant may be due to it overlapping with dysphagia. The impaired level of consciousness makes patients unable to swallow which generates the need to use a nasogastric tube leading to dysphagia. There are several unique strengths in this study. This is a first study to our knowledge designed for predicting SAP particularly for AIS patients who underwent endovascular therapy in particular. We carefully evaluated the demographic features, vascular risk factors, clinical features as well as multiple blood parameters of these patients. The generated model with risk stratification has good discrimination and calibration. Importantly, when compared with the existing A^2^DS^2^ and ISAN scores, SDL index showed the best discrimination among our study population, which indicated that SDL index might be a better way to predict SAP for AIS patients who underwent endovascular therapy. To our knowledge, this is the first study which investigated a prediction model including a blood parameter associated with stroke-induced immunodepression. We also compared the SAP patients' 3-month outcome with non-SAP patients', which indicated that SAP was significantly associated with unfavorable prognosis. What's more, all the components in the SDL index are routinely collected in the clinical work.

We believe that the SDL index will be a largely preferred model in the prediction of SAP for acute ischemic stroke patients who underwent endovascular therapy due to its well-validated, simple and cost-effective nature. This study also has limitations. First, this prediction model came from a single-center and retrospective study with a limited sample size, in comparison to other validated predictive scores such as the ISAN or the A^2^DS^2^ scores, this might generate selection bias. Worth to be mentioned, the scale has been developed and validated particularly in stroke patients underwent endovascular treatment. Therefore, its predictive value in non-treated stroke patients should be further evaluated. Second, mechanical ventilation should be better considered even though our study population based on patients who underwent local anesthesia. In addition, considering the procedures of the endovascular treatment itself, we evaluated time intervals, operational details, and recanalization rates. While some other parameters such as the length of the procedure and number of attempts to recanalization were not included in the design of this study. What's more, further studies are needed to investigate the association with the severity of the SAP.

## Conclusion

The SDL index (Stroke history = 1, dysphagia presence = 1, lymphocyte count < 1.0 × 10^3^/μL = 1) is a novel feasible tool to predict SAP in acute ischemic stroke patients who underwent endovascular treatment. This prediction model is simple and cost-effective which can be easily applied in clinical work.

## Data Availability Statement

The raw data supporting the conclusions of this article will be made available by the authors, without undue reservation.

## Ethics Statement

The studies involving human participants were reviewed and approved by the Institutional Review Board of Xuanwu Hospital. The patients/participants provided their written informed consent to participate in this study.

## Author Contributions

BZ coordinated the study, collected and analyzed the data, and drafted the manuscript. WZ, CW, LW, KK, and YD helped to modify the manuscript. CH participated in the statistical analysis. CL, JC, JD, YZ, and HC participated in the coordination of the study. XJ is the corresponding author and participated in the coordination and helped to draft the manuscript. All authors read and approved the final manuscript.

## Conflict of Interest

The authors declare that the research was conducted in the absence of any commercial or financial relationships that could be construed as a potential conflict of interest.
