# Supplementary material for: SDL Index Predicts Stroke-Associated Pneumonia in Patients After Endovascular Therapy
Source: Front Neurol. 2021 Feb 16;12:622272. doi: 10.3389/fneur.2021.622272 (PMC7921145; doi:10.3389/fneur.2021.622272)
Supplement: Supplementary file 1 [file Data_Sheet_1.docx]

**Supplementary material**

Table 1 Blood parameters evaluated in the multivariable logistic regression

|  | Adjusted OR | 95% CI | P value |
| --- | --- | --- | --- |
| Neutrophil | 1.003 | 0.866-1.162 | 0.964 |
| Lymphocyte | 0.303 | 0.124-0.741 | 0.009 |
| D-dimer | 1.034 | 0.904-1.182 | 0.628 |
| NLR | 1.050 | 0.989-1.114 | 0.111 |
| PLR | 1.003 | 1.000-1.007 | 0.082 |
| MLR | 2.678 | 0.765-9.377 | 0.123 |

Adjusted for age, gender, diabetes mellitus, atrial fibrillation, previous stroke history, systolic blood pressure, dysphagia, initial NIHSS score, GCS score and recanalization

Figure 1 Receiver operating characteristic (ROC) curve for lymphocyte count in predicting SAP.

Figure 2 Receiver operating characteristic (ROC) curves for dysphagia and stroke history in predicting SAP.

Table 2 ROC curve was used to evaluate the predictive value of lymphocyte count on SAP.

|  | AUC | 95% CI | P | Optimal cut-off value | Sensitivity | Specificity |
| --- | --- | --- | --- | --- | --- | --- |
| Lymphocyte | 0.672 | 0.605-0.738 | ＜0.001 | 1.00 | 63.6% | 62.7% |
| Dysphagia | 0.678 | 0.611-0.749 | ＜0.001 |  |  |  |
| Stroke history | 0.599 | 0.523-0.669 | ＜0.001 |  |  |  |

AUC, area under the curve; CI, confidence interval
